# Complementary therapy with Chinese aromatic herbs to promote awakening in a comatose patient: A case report

**DOI:** 10.1097/MD.0000000000039277

**Published:** 2024-08-09

**Authors:** Shi-Jia Chen, Chang-Lin Qiu, Li-Ping Zhang, Ling-Zhi Jiang, Xiao-Yu Zhao, Qun Hou, Yan Jiang

**Affiliations:** a The First School of Clinical Medicine, Zhejiang Chinese Medical University, Hangzhou, China; b Department of Neurology, The First Affiliated Hospital of Zhejiang Chinese Medical University (Zhejiang Provincial Hospital of Chinese Medicine), Hangzhou, China; c Qiu Changlin Provincial TCM Master Studio, Hangzhou, China; d Department of Rehabilitation, Zhejiang Provincial People’s Hospital, Affiliated People’s Hospital, Hangzhou Medical College, Hangzhou, China; e Department of Neurology, Zhejiang Provincial People’s Hospital, Affiliated People’s Hospital, Hangzhou Medical College, Hangzhou, China.

**Keywords:** Chinese aromatic herbs, complementary therapy, disorder of consciousness, traumatic brain injury

## Abstract

**Rationale:**

Traumatic brain injury frequently leads to prolonged coma, posing significant medical management challenges. Complementary therapies, including traditional Chinese herbal medicine, have been investigated as potential interventions in comatose patients. Chinese aromatic herbs, such as Borneolum (Bingpian), Moschus (Shexiang), and *Acori tatarinowii* rhizoma (Shichangpu), have long been believed to be “resuscitation with aromatics” based on traditional Chinese medicines theory.

**Patient concerns:**

A 16-year-old male was admitted to the intensive rehabilitation unit for further treatment due to prolonged coma and frequent seizures following traumatic brain injury.

**Diagnoses:**

Western medicine diagnosed the patient as coma, diffuse axonal injury, and epilepsy. According to traditional Chinese medicine theory, the syndrome differentiation indicates a Yin-closed disease.

**Interventions:**

According to the patient’s condition, we use the Chinese aromatic herbs as a complementary therapy.

**Outcomes:**

Following a month-long administration, the patient’s consciousness and electroencephalogram (EEG) background progressively improved. A 6-month follow-up demonstrated full arousal, though with ambulatory EEG revealing mild to moderate abnormality in the background.

**Lessons:**

The addition of Chinese aromatic herbs appears to have a beneficial effect on the patient’s consciousness and EEG background. This could be attributed to the herbs’ inherent pharmacological properties, as well as their potential to enhance the permeability of the blood-brain barrier to other drugs. This makes them a promising option for complementary therapy.

## 1. Introduction

Addressing disorder of consciousness (DoC) attributable to acute traumatic brain injury (TBI) is a complex and significant challenge.^[1]^ The clinical manifestations of DoC following acute TBI often include a state of prolonged altered consciousness, which is one of the most common and significant symptoms in neurology. DoC often initiates with coma and may progress to vegetative and/or minimally conscious states as it persists.^[2]^ The return of awareness is unpredictable, potentially taking weeks, years, or never occurring.^[3]^ Notably, seizures have been reported in approximately 17% of individuals after acute TBI,^[4]^ which adds another layer of complexity to DoC. Proper medical intervention can reduce the risk of recurrent seizures and improve overall outcomes for TBI patients. Overall, the outcomes of individuals with DoC resulting from TBI can vary widely depending on the brain networks and neuronal mechanisms.^[1]^

Currently, clinical treatment involves modern agents, hyperbaric oxygen,^[5]^ brain stimulation,^[6–8]^ and traditional rehabilitation therapy,^[9]^ etc. Moreover, adjunctive treatment methods, including music therapy,^[10,11]^ brain-computer interface,^[12]^ and rehabilitation robot,^[13]^ are gaining attention. There have been significant advances over the last decade in promoting the recovery of consciousness in TBI patients with DoC, but treatment options remain limited.^[14]^ Developing more personalized, accessible, and integrated approaches to recovery and rehabilitation is crucial. Various branches of traditional Chinese medicine (TCM), including acupuncture^[15]^ and herbal medicine,^[16]^ are employed widely.

In the past 20 years, research into Chinese aromatic herbs has garnered significant attention. Aromatherapy drugs are known for their efficacy in clearing the orifice and have a long history of treating conditions such as orifice occlusion, dizziness, phlegm obstruction, internal blockage, etc. Chinese aromatic herbs, such as Borneolum, Moschus, and *Acori tatarinowii* rhizoma, have long been recognized for their profile of “resuscitation with aromatics” and “promoting mental alertness” based on TCM theory.

The purpose of this paper is to discuss the clinical value and potential mechanism of aromatherapy by reporting a case with persistent DoC and recurrent seizures following acute TBI.

## 2. Case presentation

**Figure 1.**
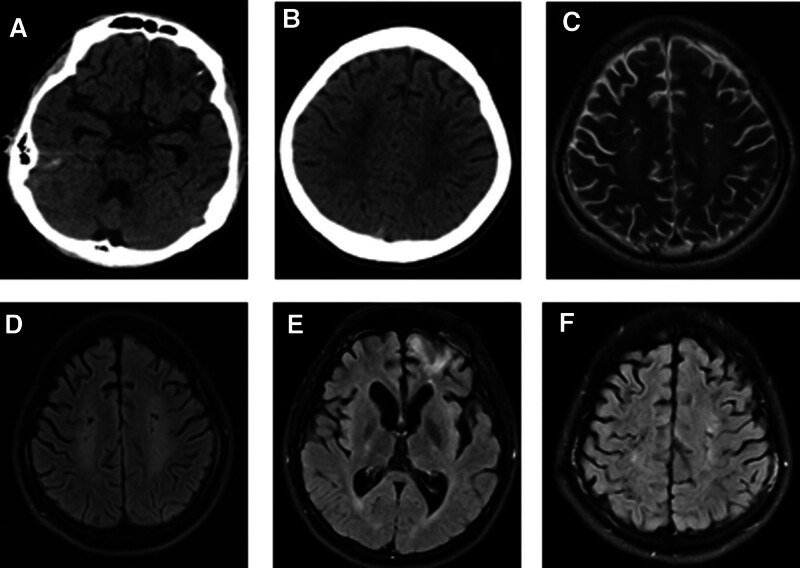
One month following the trauma, a CT scan revealed left frontal malacia (A), along with bilateral centrum semiovale hypodensity (B). Concurrently, the skull MRI indicated high T2WI signals and low T2 FLAIR signals (C and D), suggesting the presence of diffuse axonal injury. On the 6-mo magnetic resonance imaging, there was evidence of left frontal malacia, mild cerebral atrophy (E), and bilateral centrum semiovale hyperdensity (F). CT = computed tomography, MRI = magnetic resonance imaging.

A 16-year-old male patient was admitted to the local hospital after falling from a height of 3 m. At that time, the patient was conscious, felt pain in both lower limbs, and was unable to walk. Computed tomography examination revealed multiple fractures to the head, as well as subarachnoid hemorrhage. Seven hours later, the patient suddenly became unconscious with convulsions which lasted for several hours. The local hospital suspected an epileptic seizure after brain trauma and administered antiepileptic treatment, and other interventions before transferring the patient to the intensive care unit of a tertiary hospital for further care. During hospitalization, the patient experienced recurrent seizures despite the administration of several antiseizure medicines, including midazolam, valproate, levetiracetam, and clonazepam, without satisfactory effect. Subsequently, the patient developed a high fever and pneumonia, and experienced persistent coma. A brain diffusion tensor imaging at 25 days postinjury indicated diffuse axonal injury (Fig. 1). The EEG revealed severe abnormalities, characterized by dominant delta waves in the background activity, along with a few theta waves. Forty days after falling, the patient’s poor consciousness (GCS: E_4_V_1_M_2_) persisted and he was transferred to the intensive rehabilitation unit where Chinese aromatic herbs were adopted as a complementary therapy with the primary aim of promoting consciousness recovery. Before starting TCM treatment, the tongue presented a pink color, with a wet and white coat, while the pulse felt deep and slippery. According to the diagnostic methods of TCM, the syndrome differentiation indicates a Yin-closed disease. The main aromatic herbal is the Styrax (Suhexiang) pill, which comprises 15 aromatic herbs while styrax is the main component, that has been approved by the Chinese National Drug Administration, and administered at a dosage of 1 pill per time, twice daily. Additionally, the patient received a prescription for a modified Ditan decoction containing *Acori tatarinowii* rhizoma following TCM syndrome differentiation. At the same time, the powder of Moschus 0.3 g is added to the decocted herbs twice daily. After 2 weeks, the prescription was adjusted to a modified version of Changpu Daotan decoction, which still included *Acori tatarinowii* rhizoma.

**Figure 2.**
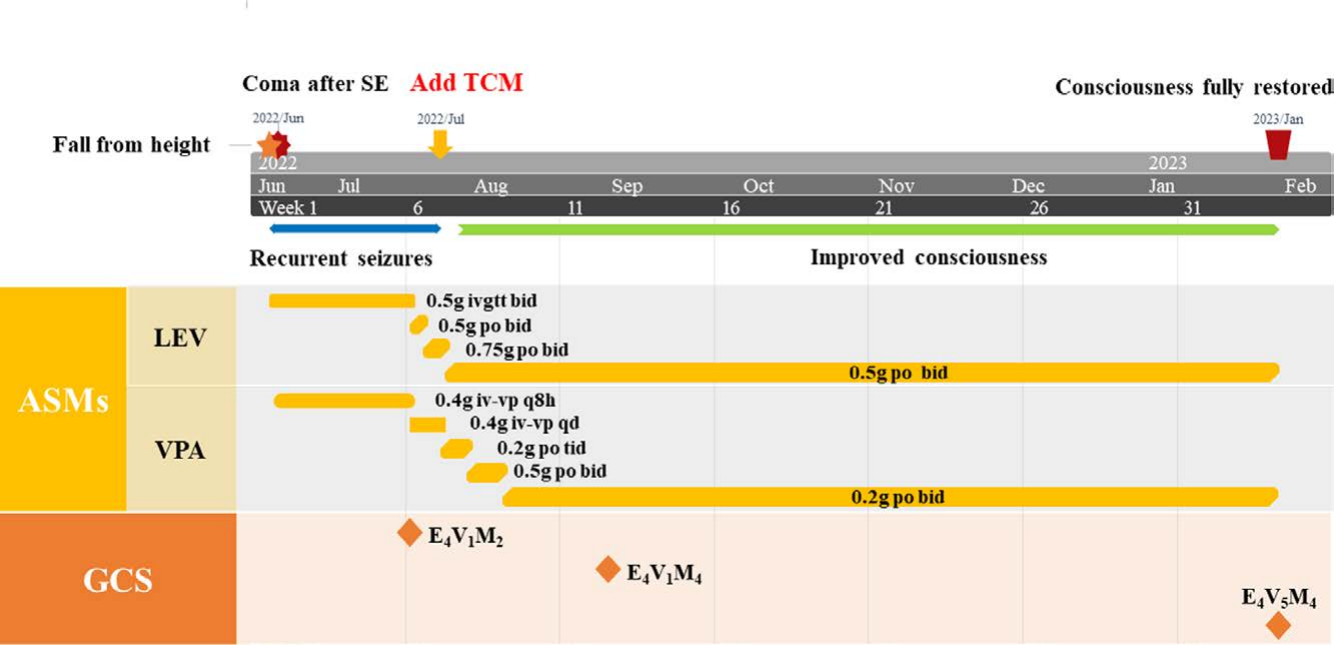
Timeline of the patient’s medication history, which shows the clinical history, antiseizure medicine treatment, and GCS score in our patient. ASMs = antiseizure medications, GCS = Glasgow coma scale, LEV = levetiracetam, SE = status epilepticus, TCM = Traditional Chinese Medicine, VPA = valproate.

**Figure 3.**
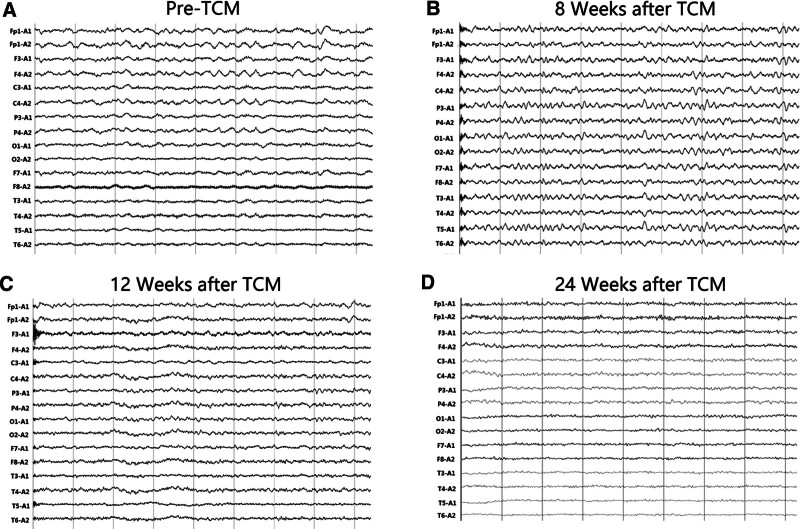
The EEG before the use of TCM showed abnormal background activity, predominantly with delta waves and some theta waves (A). After taking TCM for 8 wk, a repeat EEG showed predominantly theta waves (B). After 12 wk, there was a further reduction in slow waves compared to the EEG at 8 wk (C). At the 6-mo follow-up, the EEG still showed mild abnormalities (D). EEG = electroencephalogram, electroencephalogram, TCM = Traditional Chinese Medicine.

One month after adding TCM, the consciousness of the patient improved obviously (Fig. 2). The patient has a retracted limb response to painful stimuli. GCS score was 9. Two months after adding TCM, the EEG background of the patient improved (Fig. 3). The slow waves in the EEG background of this patient were reduced. A 6-month follow-up demonstrated full arousal, has spontaneous speech, and can repeat, name and read, though with ambulatory EEG revealing mild to moderate abnormality in the background without epileptic discharges. GCS score was 13. Magnetic resonance imaging examination at 6 months revealed left frontal lobe softening, mild brain atrophy, and bilateral hemioval center density (Fig. 1).

The patient did not report any adverse reactions during the treatment, was satisfied with the effect of Chinese aromatic herbs, and confirmed its curative efficacy.

## 3. Discussion

The study reports that the median duration of acute care for patients with DOC is about 25 days upon admission to rehabilitation and 37 days at discharge from rehabilitation.^[2]^ Furthermore, seizures occur in 27% of patients with DOC and are believed to impact the recovery of consciousness and prognosis.^[17]^ The administration of antiseizure medicines in DoC must be carefully evaluated. Some medications, like benzodiazepines or intravenous anesthetics, have the potential to worsen both consciousness and EEG background. Aromatic herbs in TCM theory serve a dual purpose by improving both consciousness impairment and epileptic seizures, making them an ideal complementary therapy.

In this case, it seems like the addition of aromatic herbs has had a positive impact on the patient’s consciousness and EEG background over the course of 1 month. The fact that the patient was completely awake during the 6-month follow-up is encouraging. However, the presence of mild to moderate abnormality in the ambulatory EEG indicates that there may still be some underlying issues that need to be addressed. This is consistent with the results reported in the literature for many patients who recover from DoC experience lifelong cognitive impairments.^[1,2]^ It would be important to continue monitoring the patient’s progress and possibly consider further interventions or adjustments to their treatment plan.

### 3.1. Aromatic herbs and TCM theory

In this case, the main components of aromatic herbs were *Acori tatarinowii* rhizoma, Styrax, and Moschus. Other aromatic herbs included Borneolum, Benzoinum (Anxixiang), *Menthae haplocalycis* herba (Bohe), *Angelicae sinensis* radix (Danggui), *Chuanxiong rhizoma* (Chuanxiong), Asari radix et rhizoma (Xixin) and *Magnoliae flos* (Xinyi). According to TCM theory, these herbs are well-known for their ability to open the orifices, disperse Xin, promote Qi circulation, dispel evil, and awaken the spirit. The therapeutic effects of aromatic herbs can be attributed to their aromatic substances, which are administered by decoction, nasal feeding, nebulization, or nasal drops. They are primarily used in the treatment of symptoms associated with “ closure disorders,” including coma, lockjaw, clenched fists, rigid limbs, and seizures, which can be caused by various pathological factors. Numerous studies have investigated the therapeutic effects of aromatic herbs in addressing consciousness disorders resulting from diverse etiologies such as cerebrovascular disease, epilepsy, and brain trauma. Noteworthily, TCM medications are usually prescribed as a combination of multiple herbs. The representative aromatic herb prescriptions, such as Angong Niuhuang pill, Suhexiang pill, Zhibao pill, and Zixue pill, have been approved by the Chinese National Drug Administration.

An imbalance between Yin and Yang in the body is an important concept in TCM theory. Yang is associated with heat and inflammation, while Yin is associated with cold and dampness. Regarding the “closure disorder,” symptoms such as facial heat, redness, labored breathing, constipation, dry tongue with a greasy yellow coating, and a taut, slippery, and rapid pulse are characteristic of “Yang closure.” On the other hand, a patient displays a pale complexion, purplish lips, excessive phlegm, chills in the limbs, and a white coating on the tongue. Moreover, a weak and slippery pulse is characteristic of “Yin closure.” Most aromatic herb prescriptions, such as the Angong Niuhuang pill, are targeted for “Yang closure,” while very few, like the Suhexiang pill, are intended for “Yin closure.” According to TCM syndrome differentiation analysis, this patient belongs to “Yin closure.” Therefore, the Suhexiang pill was prescribed along with a Chinese medicine treatment program as a complement, and this intervention yielded positive outcomes.

### 3.2. Effect in seizure, TBI, and cerebrovascular disease

Several studies have demonstrated aromatic herbs’ antiepileptic effects. Natural Borneolum exhibits an anticonvulsant effect in mice experiencing status epilepticus. As the primary medicinal and odor-contributing ingredient in natural Moschus, muscone partially inhibits seizures induced by pentetrazole.^[18–20]^ It is noteworthy to observe the potential antiepileptic effects of *Acori tatarinowii* rhizoma and its components α-asarone.^[21,22]^ *Acori tatarinowii* rhizoma demonstrates a reduction in seizure incidents in a maximal electroshock model. In rats administered with pentylenetetrazole, the *Acori tatarinowii* rhizoma decoction led to a decrease in convulsive rates.^[23]^ Additionally, α-asarone shows promise in reversing drug resistance in P-glycoprotein-mediated drug-resistant epilepsy.^[24]^

Some studies have examined the use of aromatherapy in patients with TBI.^[16,25]^ Muscone, *Acori tatarinowii* rhizoma, and Borneolum were studied for their efficacy in treating diffuse axonal injury-induced coma through nasal inhalation. A significant improvement in cerebral blood flow velocity and cerebral blood flow was observed in the study, which may be related to the enhanced rate of early awakening and late improvement.^[26]^

Aromatherapy also plays a significant role in the management of acute cerebrovascular disease. For instance, Xingnaojing, which is the injection form of Angong Niuhuang pill, may aid in the recovery from acute cerebrovascular disease. Xingnaojing, alone or combined with other medicines, had a positive effect on patients with fever, poisoning, and stroke-induced comas.^[27]^ It has been shown that *Acori tatarinowii* rhizoma decoction can reduce neurological deficits, lower levels of inflammatory factors, and improve consciousness disorders in acute cerebral infarction.^[28,29]^

### 3.3. The mechanisms of aromatic herbs

Research indicates that aromatic herbs can enhance blood-brain barrier (BBB) permeability.^[30]^ The combination of aromatic herbs with other drugs has been found to enhance the ability of those drugs to penetrate the BBB.^[31]^ This can lead to increased efficacy of the drugs in reaching the brain and exerting their therapeutic effects. It’s interesting to note that aromatic herbs have been found to have protective effects on the BBB structure in rats with cerebral ischemia-reperfusion injury.^[32]^ Borneolum exhibits an anticonvulsant effect in mice experiencing status epilepticus. It appears to be the most effective in this regard, followed by Moschus, while styrax and Benzoinum exhibit weaker effects.^[32]^ Interestingly, Borneolum exhibits an anticonvulsant effect in mice experiencing status epilepticus. It also exhibits bidirectional regulatory effects, capable of increasing physiological BBB permeability while decreasing pathological BBB permeability.^[33]^ Leveraging the properties of Borneolum exhibits an anticonvulsant effect in mice experiencing status epilepticus, a novel drug delivery system targeting the brain has been developed in conjunction with nanomaterials.^[34,35]^

The specific mechanism of how aromatic herbs achieve this enhancement is an active area of research and study. Various aromatic substances demonstrate diverse mechanisms of action, with Borneolum being the most extensively studied. Borneolum and α-asarone as adjuvant agents for improving the BBB permeability of puerarin and tetramethylpyrazine by activating adenosine receptors.^[36]^ Borneolum regulation of the permeability of the BBB might be related to the increased expression of intercellular cell adhesion molecule-1.^[37]^ Other mechanisms including Borneolum bidirectional regulation are related to down-regulate P-gp, inhibiting the expression of IL-1β and MMP-9, nitric oxide pathway, etc.^[33,38]^ Styrax treatment inhibits caveolae-mediated transcytosis at BBB in the focal stroke model of rats.^[39]^

Besides modulating BBB permeability, other mechanisms include modulating inflammatory responses, regulating central excitatory and inhibitory neurons and neurotransmitters,^[40,41]^ improving local microcirculation, and enhancing cerebral metabolism.^[42]^

### 3.4. Limitation

This is a single case report and lacks a control group. In addition, the efficacy of Chinese aromatic herbs alone in treating this disease cannot be adequately demonstrated when combined with conventional treatment. Hence, there is a need for more detailed controlled clinical studies to be conducted.

## 4. Conclusion

In conclusion, incorporating TCM, particularly Chinese aromatic herbs, into standard rehabilitation protocols offers a promising approach to enhancing consciousness recovery and neurological function in patients with TBI, particularly those experiencing seizures. Future research should focus on elucidating the precise mechanisms of action of aromatic herbs, optimizing treatment regimens, and conducting large-scale clinical trials to validate their effectiveness in improving outcomes for individuals with DoC.

## Acknowledgments

The authors acknowledge the help of the patient and his family in consenting for publication.

## Author contributions

**Data curation:** Shi-Jia Chen, Yan Jiang.

**Writing—original draft:** Shi-Jia Chen.

**Investigation:** Chang-Lin Qiu, Li-Ping Zhang, Lin-Zhi Jiang.

**Methodology:** Chang-Lin Qiu, Li-Ping Zhang.

**Project administration:** Ling-Zhi Jiang.

**Formal analysis:** Xiao-Yu Zhao, Qun Hou, Yan Jiang.

**Conceptualization:** Qun Hou.

**Supervision:** Yan Jiang.

**Writing—review & editing:** Yan Jiang.
